# Moving beyond depression screening: integrating perinatal depression treatment into OB/GYN practices

**DOI:** 10.1017/S1463423618000099

**Published:** 2018-02-12

**Authors:** Christina Terrazas, Lisa S. Segre, Cheryl Wolfe

**Affiliations:** 1 Rush University and Medical Center, Rush Medical Associates, South Chicago, IL, USA; 2 College of Nursing, University of Iowa, Iowa City, IA, USA

**Keywords:** ACOG Recommendations Depression Screening, depression, Listening Visits, OB/GYN practice, postpartum, pregnancy

## Abstract

In 2015, the American College of Obstetricians and Gynecologists issued a recommendation to screen women for depression and anxiety symptoms at least once during the perinatal period. Nevertheless, many identified women will not receive care from a behavioral health specialist. Listening Visits (LV), developed for delivery by nurses and validated in the United Kingdom, have recently been evaluated in a US-based randomized controlled trial (RCT) which recruited research participants from three home-visiting programs and an urban OB/GYN practice. RCT results indicated clinically and significant improvement in depression symptoms. To bridge the gap between evidence and practice, and based on experiences garnered at the OB/GYN site during the RCT, this development paper proposes a strategy for implementing depression screening and LV into routine clinical care in this practice setting.

Untreated perinatal depression can cause substantial maternal and child morbidity. A confidential enquiry into maternal deaths in the United Kingdom indicated that suicide is the leading cause of maternal mortality, accounting for 28% of maternal deaths (Oates, 2003). Similarly, among US women of childbearing age, depression is the leading cause of non-obstetric hospitalizations (Jiang *et al*., 2000). Impoverished women are particularly at-risk (Segre *et al*., 2007). Depression during pregnancy is of special concern for OB/GYN physicians because of its possible impact on fetal and newborn outcomes, such as intrauterine growth restriction, pre-term labor and delivery, and low infant birth weight (Grote *et al*., 2010). In response, American College of Obstetricians and Gynecologists (ACOG) recommends screening women for depression and anxiety symptoms at least once during the perinatal period, if there is a means to provide follow-up evaluation, and if needed, treatment (ACOG, 2015). Yet, emotionally distressed women, particularly those with limited financial resources, face numerous barriers which prevent them accessing treatment from a behavioral health specialist, including unrecognized depression/anxiety symptoms, stigma associated with mental illness, and logistical difficulties (Dennis and Chung-Lee, 2006).

Listening Visits (LV) are an evidence-based depression treatment that were developed in the United Kingdom for delivery by health visitors for mothers with mild to moderate symptoms (Holden *et al*, 1989). Recognizing the potential of LV to address treatment barriers among impoverished mothers in the United States, a four-site randomized controlled trial found LV to be effective in three US home-visiting programs as well as an OB/GYN practice setting (Segre *et al.*, 2015).

Critical gaps remain between the verification of evidence practices, like LV, as effective and their subsequent implementation into broad clinical practice (Tseng, 2012). In the field of implementation science, knowledge translation is the process of moving evidence-based practices from the research phase into clinical practice (Leng *et al*., 2007). The knowledge translation framework identifies three general categories of implementation barriers: (1) knowledge – lack of familiarity with a new practice; (2) environmental – lack of time, resources or organizational constraints; and (3) attitudes – uncertainty about the value of the intervention (Leng *et al*., 2007). To inform the development of LV into primary health-care settings (Bryar and Kendall, 2001), this paper addresses each of these three barriers with respect to implementing LV in an OB/GYN practice. Specifically, following a description of LV to increase awareness, we address organizational barriers by outlining the steps of implementing LV during the RCT. At each implementation step, we identify gaps that would result from the removal of research resources, and suggest strategies for addressing these gaps in clinical settings. This proposed protocol for implementing depression screening and LV into the clinical practice of an OB/GYN practice is depicted in Figure 1. Finally, to illustrate the value of LV, a case presentation of LV in the OB/GYN clinic is provided (Box 1).

**Figure 1:**
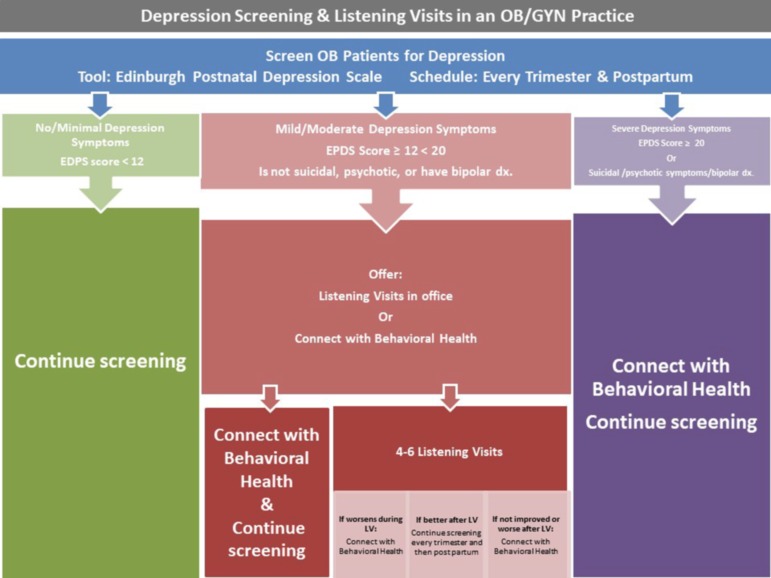
Proposed protocol for depression screening and Listening Visits in an OB/GYN practice.

Box 1 A Case Presentation of Listening Visits in an OB-GYN office

**Patient Background and Initial Screening Score** C.S., a 25-year G2P0010, married female, presented to the Physician Assistant for LV. On initial EPDS, administered during the second trimester, C.S. scored a 13. C.S. denied any personal or family history of depression. Her OB history was significant for fetal demise, which was noted on ultrasound at 13 weeks. However, during this pregnancy, C.S. had an uncomplicated prenatal course. She had a 20-week structural survey OB ultrasound that was normal with the exception of a low-lying placenta which had resolved by her follow-up ultrasound. Yet during this pregnancy, C.S. struggled with significant anxiety and was preoccupied with the thought of fetal demise. She reported difficulty sleeping due to thoughts and dreams of having another miscarriage. **Listening Visits** Over the course of eight weeks, C.S. attended five of the six scheduled LV sessions at CWHC OB/GYN office. Her individual LV sessions, about 45-minutes duration, were held weekly but separate from her prenatal visits. C.S. welcomed the opportunity to share her thoughts and concerns in the five sessions that she attended. A brief summary of each session’s focus is provided below. 1. C.S. was quick to address her fear of losing her baby and shared that these feelings did not let her sleep at night or function well at work. 2. C.S. discussed the lack of support from her husband, who she felt was annoyed by her anxiety of having another miscarriage. Therefore, she felt the need to keep her feelings to herself. 3. C.S. indicated that she was coping better because of the opportunity to talk about her fears during the LV sessions. She said she was no longer keeping her feelings “bottled up” and felt relief and less anxiety after the sessions. She still would not address her feelings with her husband. 4. We used collaborative problem solving. Specifically, C.S. identified some of her specific concerns, wrote them down, and ranked them in order of importance for her. She then decided which concern she would tackle first-- her fear of miscarriage-- and listed possible solutions. At this point, she was far enough along in her pregnancy that she could feel the baby move. She proposed that, to reassure herself and reduce her anxiety, she could periodically check for the baby’s movement. This solution also aligned with recommendations from her OB physician. She said she would only do this extra surveillance during times she felt anxious. Other solutions that she suggested included praying at home and at church. A second goal identified in the problem-solving session was her desire to rebuild her relationship with her husband. She decided to do this by setting aside time for “mini dates” where they would go for a walk together, go on a dinner date, or just watch a favorite show together. 5. C.S. was excited to say the week had passed with less anxiety and that she and her husband had a good conversation over dinner one night. She let him talk about his day and said that was a good distraction for her. They ended the day by watching a movie on the couch. C.S. indicated that this interaction gave her a sense of security. She expressed she eventually would like to discuss with him why she had been so withdrawn. **Post-LV Assessment** C.S. had a final EPDS score of seven and gave positive feedback about how LV helped her to cope with her anxiety, and that she was glad she did not have to take depression medication. C.S. said, “*after my previous miscarriage I was really afraid and Christina really listed to me, [she]let me hear the baby’s heartbeat, answered questions and helped me with my pregnancy concerns. I felt comfortable. It was nice to talk with someone who wouldn’t judge me*”.

## Listening Visits: development and empirical support

In the United Kingdom, public-health nurses, called health visitors, are central to maternal and newborn postpartum care. Universal services delivered by health visitors include an antenatal appointment around the 28th week to educate pregnant women about health-visiting services, the new birth home visit at 10–14 days postpartum, the six to eight-week postnatal contact in the home or in the clinic to review infant and maternal well-being, and staffing child advice clinics. In conjunction with the development of the Edinburgh Postnatal Depression Screening Scale (EPDS), used by health visitors to identify maternal depression (Cox *et al*., 1987), LV were concomitantly developed for health visitors as a first-line treatment approach for postpartum depression (Holden *et al*., 1989). The goal of the listening sessions is to gain a genuine understanding of a woman’s situation and then to work with her collaboratively to identify problems and implement solutions. Specifically, in a series of weekly listening sessions conducted in the home, women have the opportunity to discuss issues troubling them as a way to relieve stress. In 2007, substantial empirical support from European trials prompted the British *National Institute for Clinical Excellence* to recommend LV as evidence-based treatment for postpartum women with mild to moderate depressive symptoms (British Psychological Society, 2007).

Because LV can be provided by non-mental health specialists, this intervention has significant potential to overcome the barriers that prevent many US women from obtaining treatment from a behavioral health specialist, particularly at-risk, low-income women (Segre *et al*., 2007). However, unlike the United Kingdom where health visitors care for both the mother and newborn throughout the first year, in the US postpartum maternal care is limited to the six-week postpartum visit and provided by an OB/GYN specialist. While screening for depression is now required (ACOG, 2015), an elevated depression symptom score typically results in a referral to a behavioral health specialist and many barriers prevent women from accessing or receiving this care. Utilizing point-of-care providers, like home visitors or clinic-based advance practice providers, seemed to be a good way to address many of the barriers preventing women from receiving care. However, given the inherent differences between the UK and US healthcare systems in provision of maternal postpartum care, it was first necessary to obtain local empirical support for LV.

To evaluate LV locally, the second author conducted a four-site randomized controlled trial in three home-visiting programs in Iowa and the OB/GYN practice that is the focus of this development paper. The method and results of the RCT are fully described elsewhere (Segre *et al*., 2015). Briefly summarized here, women with elevated depression symptom scores on the EPDS (Cox *et al*., 1987) were randomized on a 2 to 1 ratio to receive LV immediately (*n*=41), or after an eight-week delay during which time the received usual care (*n*=25). At the eight-week assessment, the results indicated that 36% of women in the LV group, and 14% of women in the delayed control group experienced clinically significant improvement from the baseline assessment on the Hamilton Depression Rating Scale (Segre *et al*., 2015). Further in the follow-up phase of the RCT, among those who received LV immediately improvement in mood was sustained over an eight-week follow-up and replicated among those in the delayed control group after they received LV sessions (Brock *et al.*, 2017). Importantly, among this group of depressed impoverished mothers of young children, who often to not want to or cannot see a behavioral health specialist, results of a qualitative inquiry indicated that they valued LV as delivered by their point-of-care provider (Orengo-Aguayo and Segre, 2016).

## Implementing Listening Visits in an office setting

Early identification of perinatal depression is crucial in the development of treatment plans for this potentially devastating illness. The integration of LV into the OB/GYN practice offer health-care providers an excellent tool for the management of mild to moderate depression during pregnancy and postpartum period. To create a protocol for implementing depression screening and LV in an OB/GYN practice (Figure 1), we divided the process that we used during the RCT (labeled ‘research setting’ below) into a series of steps. At each step, we also propose how to adjust for the removal of research resources to implement depression screening and LV in an OB/GYN practice (labeled ‘clinical setting’ below).

### Step 1: selecting the site: OB/GYN practice

#### Research setting

Recruitment to the RCT was initially limited to one home-visiting program in Iowa but because of lagging recruitment rates, additional sites were required. *Chicago Women’s Healthcare* (CWHC) is a small, full-scope OB/GYN private practice located near an urban medical center on the south side of Chicago. The patient base is diverse and represents several ethnic communities. Patient household income, education and health literacy levels are typical of OB/GYN practices in this part of the city. The location of CWHC as well as the fact that the practice was not a home-visiting program distinguished this site from the three home-visiting sites of the RCT.

#### Clinical setting

There is no ideal size or patient demographic of an OB/GYN practice best suited for LV. However, it is essential that the office or clinic have engaged staff and health-care providers. The overall goal is to provide a non-intimidating environment for the patient to schedule and receive her listening sessions. There should be a designated room for LV to occur, away from the rest of the patient rooms is most desirable but not always possible. The practice should also have a perinatal depression screening process already in place and ongoing reassessment of the LV workflow.

### Step 2-selecting the LV provider

#### Research setting

CWHC employs one full-time OB/GYN physician, a physician assistant, two medical assistants, a receptionist, and a practice manager. During the RCT, the physician assistant (first author), provided all the LV sessions to patients at this RCT site. In the United States, a physician assistant is a licensed health professional who has completed a 26-month, post-university educational program that requires the same prerequisite courses as medical schools. Upon completion, physician assistants must pass a national certifying exam to be licensed to practice. In addition, they must obtain an additional license to be able to practice in their state.

#### Clinical setting

In an OB/GYN practice, a bachelor’s level registered nurse, social worker, or a physician assistant could provide LV. Ability to bill for time may influence choice of provider in each setting.

### Step 3: providing LV training

#### Research setting

The LV curriculum is comprised of three parts: education about perinatal depression, introduction to LV, and LV skills training. In the RCT, education about perinatal depression was already in place in association with their established depression screening protocol. To familiarize key CWHC personnel with the LV intervention, the physician and physician assistant attended a brief presentation describing the history of LV development and the evidence base for LV in European trials. To learn LV-specific skills, the physician assistant also attended a one-day workshop on LV delivery. This LV-skills training covered introducing the intervention and training in the use of LV skills, including empathic responding and problem solving. Skills training emphasizes that an LV provider does active listening for the early sessions. In later sessions, once the LV provider has a clear understanding of the patient, the focus turns to collaborative problem solving. Here the LV provider has the patient identify areas that are causing anxiety/concern. The patient is then requested to rank these concerns in order of importance. The remaining visits are used as problem-solving sessions, with a final session to summarize.

#### Clinical setting

To educate staff about perinatal depression, there are three widely available low-cost educational resources. First, Postpartum Support International (PSI) is a nonprofit organization whose mission is to increase awareness of emotional changes in the perinatal periods. The PSI website provides extensive information for women and educational resources for health-care professionals, including a complimentary 90-minute webinar on maternal mental health: http://www.postpartum.net/. Second, the ACOG website offers an array of information concerning depression and postpartum depression, including links to the Guideline for Perinatal care (for providers) and postpartum depression (for patients) https://www.acog.org/Womens-Health/Depression-and-Postpartum-Depression. Third, *Beyond the Blues: Understanding and Treating Prenatal and Postpartum Depression* (Bennett and Indman, 2003), which is available in both English and Spanish, educates readers about mood disorders in the perinatal period and treatment options. The concluding chapter provides a list of helpful resources.

The introduction to LV and LV-skills training require attending a specialized workshop. While not broadly available currently, LV training is offered for clinical implementation purposes in both the United Kingdom (Hanley, 2015) and in the United States from the second author (Colorado Maternal and Child Health Program, 2013). Use of a train-the-trainer model for LV training could significantly increase the availability and sustainability of this approach. Exploring the effectiveness of this training model is an important direction for future research.

### Step 4: identifying women appropriate for LV

#### Research setting

Before the RCT and as part of routine clinical practice, all pregnant CWHC patients were screened for depression every trimester and at the postpartum visit, using the EPDS and a cutoff score of 12 or above (Cox *et al*., 1987). Consistent with this established clinical practice, during the RCT, women with an EPDS score of 12 or above, who were not already seeing a behavioral health-care specialist were invited to participate in the RCT. Those who were not interested in the RCT were referred to a behavioral health specialist. Women, who opted for LV in the RCT, were then screened for a second time by the research team to identify women whose symptom profile was not appropriate for LV. Such exclusions included, suicidality (ie, a rating of 3 on item #10 of the EPDS), symptoms of psychosis, indications of active substance use, or a diagnosis of bipolar disorder putting them at risk for postpartum psychosis. Ineligible women were referred directly to behavioral health-care specialist.

#### Clinical setting

In accordance with early NICE guidelines (British Psychological Society, 2007), the LV intervention is suitable for women with mild to moderate depression symptoms. In routine clinical practice, the EPDS can be used to identify symptom severity for both the lower (mild) and upper bounds (moderately) of this recommendation. For the mild range (or lower bound), results of a large screening trial suggest that a score an EPDS score of 10 should be considered. Specifically among the 826 women with an EPDS score of 10 or higher, 90.8% had symptoms meeting the criteria for Major Depressive Disorder as assessed by diagnostic clinical interview (Wisner *et al*., 2013). For the moderately severe range (or upper bound), an EPDS score of 20 or above is suggested. Specifically in a study in which women completed both the EPDS and the Beck Depression Inventory (BDI), scores of 20 or higher on the EPDS corresponded to BDI scores in the severely depressed range (McCabe-Beane *et al*., 2016).

To replace the diagnostic interview used by the research team to identify symptoms profiles not appropriate for LV, we propose to embed this assessment within the routine patient history to assess for bipolar disorder diagnosis, past and current substance abuse. Many Electronic Health Record systems have tools that facilitate screening these types of screening so that patients can be referred to a behavioral health specialist. Finally, active suicidality can be determined using the same strategy employed by the RCT research team: a rating of three on item #10 of the EPDS.

### Step 5: delivering LV sessions

#### Research setting

To implement LV in the OB/GYN practice during the RCT, a workflow was established to schedule the patients for a set time in the room designated for the LV, and a phone call system was put in place to remind the patient of the LV session and inquire if childcare would be needed for that time. In addition, the physician assistant had allocated time on her schedule to ensure that the LV could be completed without her feeling rushed. This entire process was reviewed with the staff on a regular basis, any concerns were addressed immediately, and adjustments made accordingly (ie, patient may prefer text message to phone call for reminder). Eligible CWHC patients received up to six, weekly, 50-minute LV sessions, in addition to standard physical-health prenatal or postpartum visits. The LV sessions took place in CWHC office, which was familiar; and were scheduled either separately from their prenatal care appointment or on the same day in an additional timeslot. For illustrative purposes, a case example of LV, as provided by the physician assistant at the CWHC office, is provided in Box 1.

#### Clinical setting

In clinical care, a workflow similar to the one described for the RCT trial should be used. However, the number and length of sessions is open for development. In clinical practice in the United Kingdom, women are offered four listening sessions, ~30–40 minutes duration, with the option of adding two additional sessions. This same approach might be considered in the United States. Of note, however, several patients who had participated in LV in CWHC commented that they would have enjoyed more than the six sessions they received. Choice of LV provider, number and length of sessions will need to be determined by staff availability and level of reimbursement.

### Step 6: assessment

#### Research setting

During the RCT, the research team assessed participants before and after LV to determine whether the intervention resulted in improved mood scores. At the completion of LV, women with elevated depression scores were referred directly to a behavioral health specialist.

#### Clinical setting

In routine clinical practice, the EPDS could be used to assess the woman’s mood, both before and after LV. As indicated in Figure 1, women with sustained elevated EPDS scores after LV could be offered a referral to a behavioral health specialist. We anticipate that a positive experience with LV would facilitate her accepting this referral.

### Step 7: billing and coding for Listening Visits

#### Research setting

Several elements of LV provision in this OB/GYN practice were fiscally supported by RCT funding, including time of physician assistant to provide LV sessions, daycare that was provided by an office secretary when needed, and transportation costs to and from each LV session.

#### Clinical setting

Replacing RCT fiscal support is perhaps the most vexing, yet critical challenge to integrating LV into routine obstetrical care. At first glance, this challenge may seem insurmountable and may lead to the premature discard of this innovative redesign of mental-health services. However, this issue can be approached from different viewpoints. For instance, financial constraints have been successfully addressed in a recent statewide implementation of LV in the State of Iowa’s maternal health clinics which serve at-risk, impoverished mothers through the provision of home-visiting services (Colorado Maternal and Child Health Program, 2013). Here, the Iowa Department of Public Health obtained Medicaid billing numbers for LV provided by a nurse or social worker. While use of this LV-related Medicaid billing number is limited to the State of Iowa, this local implementation into routine clinical practice provides one example of how this difficult fiscal challenge was successfully addressed.

The ACOG Committee on Health Economics and Coding lists several ICD-10 codes that are potentially reimbursable when providing services for patients with perinatal depression (Tyler *et al*., 2016). These codes are included in the group, ‘R45- Symptoms and signs involving an emotional state.’ Specifically, codes such as R45.0 – Nervousness, R 45.1 – Restlessness and agitation, R45.2 – Unhappiness, and R45.81 – Low Self-Esteem may be appropriate to use when coding for LV. While there are additional ICD codes for mental-health disorders, these codes will most likely only be reimbursable for behavioral health specialists. The actual reimbursement amount depends upon a variety of factors including, negotiated rates between the health-care provider(s) and insurance entity, appropriate visit documentation, Level of Visit [evaluation & management (E&M) code], and geographic region.

As advanced practicing providers (eg, physician assistants and nurse practitioners) become more integrated into physician practices, their services provide another avenue for implementation of LV in a private office setting. Patients can be scheduled to see the OB-physician and then see the physician assistant or the nurse practitioner on the same date for an LV session. Here the LV session should be billable as a separate E&M code. While this scenario does not address all concerns, it does tackle the transportation issue of multiple trips by the patient to the office as well as charge capture for LV.

In addition to exploring options for financial reimbursement, broad consideration should also be given to the overall value of LV in the office setting. For example, in one randomized trial and economic evaluation which compared outcomes of usual care with two health visitor delivered interventions (Listening Visits and Cognitive Behavioral Therapy), the total time with the health visitor was less in the intervention group at the six-month assessment (Morell *et al*., 2009). Specifically, although extra time was devoted to the delivery of an intervention, the average time spent by health visitors over a six-month evaluation period was less in the two intervention groups (185.6 minutes) than in usual care group (202.4 minutes). Providing this type of first-line intervention did not result in increased costs and resulted in a modest decrease in total time with health visitor. In addition, the expense of not identifying and addressing perinatal depression can be immense. Multiple trips to the emergency room for vague symptoms, additional phone calls and visits to the office, work absences and other concerns may be secondary to undiagnosed depression in pregnancy and postpartum period. Thus, an important direction for development work will be to assess such indirect cost saving outcomes associated with LV.

## Discussion and conclusion

During their participation as a site in RCT of LV, the staff of CWHC observed that adding LV as an option in the management of perinatal depression strategically overcame many barriers pregnant and postpartum patients faced with the treatment of depression. These obstacles included mistrust of behavioral health specialists, the stigma and shame often associated with receiving depression treatment, and logistical barriers to receiving care. CWHC patients were acquainted with the physician assistant who delivered LV because she had also provided some elements of their prenatal care, so an offer of listening sessions from this familiar provider leveraged an established, trusted relationship. In addition, seeing this health-care provider for listening sessions did not evoke feelings of embarrassment often associated with talking with a behavioral health specialist. Furthermore, patients were familiar with the location of the office, eliminating another potential barrier. Finally, the improvement in mood described in the case presentation in Box 1 represents a typical experience of LV recipients in the RCT.

Implementing ACOG’s recommended screening of women for depressive and anxiety symptoms raises the inevitable issues of identifying treatment referrals and, most importantly, coming to terms with the fact that even when a referral is made, many women will not access specialist care. LV provide an effective first-line depression treatment for perinatal patients with mild to moderate depression symptoms and women value this approach. Some challenges do remain in the implementation of such an integrated model of depression management into routine clinical practice in the OB/GYN practice setting. Key among those challenges is finding time in a busy practice, providing for childcare, fiscal reimbursement and the limited availability of LV training. Suggestions for overcoming these challenges have been the focus of this development paper. Despite potential implementation challenges, LV integrated into an OB/GYN practice settings provides an evidence-based approach to addressing maternal depression early on. This model is thus an innovation worthy of further consideration.
